# Sneezing and Shivering

**Published:** 1884-06

**Authors:** 


### SNEEZING AND SHIVERING.

NATURE'S provision against the consequence of a "chill," and for the prevention of a "cold" are sneezing and shivering. A violent fit of sneezing often saves a chilled body the consequences of the nerve depression or "shock" to which it has been subjected, and this shock may in its first impression be very limited in its area; for example, the small extent covered by a draught of cold air rushing through the crevice of a door or window. The nerve centres are roused from their "collapse" by the commotion or explosive influence of the sneeze. If sneezing fails nature will try a shiver, which acts mechanically in this way. If this fails, the effects are likely to be very serious and bad consequences may ensue. The cold is slight when sneezing suffices to recover the nervous system quickly from its depression; and grave when even strong shivering fails to do so. In case of chill, with threatened cold, sneezing may be produced by a pinch of snuff of any kind. This is how some of the vaunted "cures" of cold by snuff are brought about. Brisk exercise may also ward off the attack.—*London Lancet.*

Perhaps not one the events recorded in Scripture has given rise to more fruitless controversy than the alleged act of Joshua commanding the sun and the moon "to stand still," in the memorable rout of the five Kings, and that these bodies obeyed him. Did this miracle occur in the way the ordinary translation reads and as the most of the orthodox believe it did? An able writer in the *Church Quarterly Review* endeavors, with a high degree of probability, to maintain that what the Israelitish leader prayed for was not that the snn and moon might "stand still" but they might "be silent"—that is to say, "cease to shine"—*dom shemesh,* as the Hebrew text has it. A storm of hailstones was the principal cause of the defeat of the allied Kings. Joshua, finding that the storm and darkness by which it was accompanied did more toward the overthrow of the enemy than his own troops, naturally prayed that the darkness might continue until the utter ruin of the foe was accomplished. The formidable astronomical objections to the miracle are thus at once removed by a simple philological discovery.

To disinfect a room, place an ordinary house shovel over the fire until it become thoroughly hot (but not *red hot*); then take it to the *centre* of the room and pour on the shovel an ounce of No. 4 or No. 5 carbolic; lean the shovel *so that no fluid can fall to the floor,* and the carbolic will be readily given off in vapor sufficient to fill an ordinary room. This will disinfect the air of the room, and as genuine carbolic (more properly called phenol or phenylic alcohol) is not a mineral corrosive acid, the vapor will in no way injure pictures, metals, or fabrics.

